# *Ex Vivo* Efficacy of SAR442257 Anti-CD38 Trispecific T-cell Engager in Multiple Myeloma Relapsed After Daratumumab and BCMA-targeted Therapies

**DOI:** 10.1158/2767-9764.CRC-23-0434

**Published:** 2024-03-12

**Authors:** Alana L. Keller, Lauren T. Reiman, Olivia Perez de Acha, Sarah E. Parzych, Peter A. Forsberg, Peter S. Kim, Kamlesh Bisht, Hongfang Wang, Helgi van de Velde, Daniel W. Sherbenou

**Affiliations:** 1 Division of Hematology, Department of Medicine, University of Colorado Anschutz Medical Campus, Aurora, Colorado.; 2 Sanofi R&D, North America, Cambridge, Massachusetts.; 3 Oncology Development, Sanofi, Brussels, Belgium.

## Abstract

T cell–engaging antibodies (TCEs) are showing promising efficacy in relapsed/refractory multiple myeloma, even in patients that relapsed after B-cell maturation antigen (BCMA)-targeted therapy. Patients with multiple myeloma may have compromised T-cell health unaccounted for by preclinical models. Here, we use Myeloma Drug Sensitivity Testing (My-DST) for *ex vivo* measurement of anti-multiple myeloma cytotoxicity for the trispecific CD38/CD28xCD3 TCE SAR442257 through activation of the patients’ own endogenous T cells to inform clinical development of the compound in multiple myeloma. My-DST incubates primary mononuclear cells in humanized media for 48 hours followed by flow cytometry for multiple myeloma cell viability with or without drug treatment. SAR442257 was tested on 34 samples from patients with multiple myeloma across disease settings. Potential biomarkers, T-cell dependence, and degranulation were assessed. SAR442257 was effective at low dose in My-DST cultures. High *ex vivo* response rates were observed in primary aspirates taken from patients with multiple myeloma at diagnosis, with modestly reduced response in multiple myeloma recently treated with anti-CD38 mAbs. SAR442257 was highly effective in patients relapsing after BCMA therapy. The CD38/CD28xCD3 trispecific format was substantially more effective than a conventional bispecific CD38/CD3 antibody format and CD38 mAbs. Anti-multiple myeloma cell cytotoxicity was dependent on the presence of endogenous T cells. Surface CD38 expression was the strongest biomarker of TCE response. My-DST is capable of measuring T cell–dependent killing using the multiple myeloma patient's own bone marrow–derived T cells. SAR442257 shows promise for multiple myeloma and may be best suited for patients declared resistant to both CD38 mAbs and BCMA-targeted therapy.

**Significance:**

This study introduces the use of My-DST to measure and characterize sensitivity to anti-CD38 T-cell engager SAR442257 in primary samples using matched endogenous T cells. Preclinical testing in samples from patients with diverse treatment history supports further testing in post-chimeric antigen receptor T-cell multiple myeloma.

## Introduction

Multiple myeloma is a plasma cell malignancy with debilitating complications including renal failure, immunodeficiency, anemia, lytic lesions, and fractures. Unfortunately, multiple myeloma is incurable, with most patients undergoing multiple relapses and treatment with sequential lines of therapy until they develop resistance to the standard toolbox of approved therapies. In particular, immunotherapy has changed the outlook for patients with multiple myeloma, because the approval of anti-CD38 mAbs daratumumab (Dara) and isatuximab (Isa). Typically, relapsed refractory multiple myeloma (RRMM) exhibits more aggressive behavior, leading to a shortened survival for patients who have become resistant to CD38 mAbs (1). To address this, two chimeric antigen receptor T (CAR-T) cell therapies targeting B-cell maturation antigen (BCMA), idecabtagene vicleucel (ide-cel), and ciltacabtagene autoleucel (cilta-cel), were FDA approved from 2021 to 2022 as a new class of therapy for patients with at least four prior treatment lines (2, 3). The overall response rate for cilta-cel was 98% and 73% for ide-cel, and the median progression-free survival was 9 months for ide-cel and not yet reached at 28 months for cilta-cel (2, 3). Despite these CAR-Ts’ impressive and deep responses, neither appears to be curative.

Recent advances in treating RRMM have focused on bispecific T cell–engaging antibodies (TCEs). TCEs are off-the-shelf antibodies that typically bind endogenous T cells using an anti-CD3 binding domain, while another domain engages a multiple myeloma–specific antigen. This dual-binding creates an artificial immunologic synapse sufficient to induce T-cell degranulation and multiple myeloma cell cytolysis (4). TCEs have demonstrated success in treating RRMM, exemplified by the recent FDA-approvals of BCMAxCD3-binding TCEs teclistamab and elranatamab, and GPRC5DxCD3-binding TCE talquetamab for patients who have received at least four prior lines of therapy (5–7). The efficacy of TCEs makes them an attractive option for late-stage disease, especially patients refractory to BCMA CAR-T multiple myeloma. However, despite impressive overall response rates to TCEs in patients with triple-class refractory multiple myeloma, responses are less common among patients who have receiving prior BCMA therapies (5, 7, 8). Therefore, treatment options with T-cell engagers targeting alternative antigens for patients’ post-BCMA therapy may prove more effective in some patients.

There are a growing number of TCEs in development against non-BCMA targets. One such agent, SAR442257, is a trispecific TCE that binds CD38 on multiple myeloma cells, CD3 on T cells and CD28, which is expressed on T cells and some multiple myeloma cells (9, 10). Targeting CD38 is not new in the multiple myeloma field, as CD38 mAbs Dara and Isa are commonly given in earlier lines of therapy (11, 12). SAR442257 has shown promise against multiple myeloma cell lines both *in vitro* and *in vivo*, but efficacy in primary RRMM has yet to be examined (10). As many patients with RRMM are refractory to CD38 mAbs, understanding the efficacy of SAR442257 will be critical post-anti-CD38. Some clinical studies have shown short-term success from retreatment with anti-CD38 mAbs when patients underwent a “washout” period from Dara after first relapse (13, 14). An anti-CD38 TCE like SAR442257 may be more effective in the post-anti-CD38 setting, particularly in post-BCMA patients with longer washout periods from anti-CD38 mAb treatment. Here we present data detailing the activity of SAR442257 against primary multiple myeloma cells of varying treatment history using the patient's own endogenous T cells.

## Materials and Methods

### Patient Samples

Multiple myeloma bone marrow (BM) and peripheral blood aspirates were obtained from patients at the University of Colorado Cancer Center following written informed consent from all patients and Institutional Review Board approval. Study was performed following the guidelines of the Declaration of Helsinki. Total BM and peripheral blood mononuclear cells (MNC) were isolated and cryopreserved as described previously (15). Patient age and gender was removed to deidentify primary samples. Treatment history of each patient is available (Supplementary Table S1). Aspirates with detectable multiple myeloma cells were included from both sexes as available.

### T-cell Degranulation Assays

Primary multiple myeloma aspirate MNCs were cultured with or without 1 nmol/L SAR442257 in the presence of 1 µL of GolgiStop (BD, added 2 hours prior to stain) per 3 mL of culture for 48 hours. Cells were washed and stained with anti-human CD107a-PE (H4A3) and anti-CD107b-PE (H4B4) and measured via flow cytometry.

### Myeloma Drug Sensitivity Testing

Patient samples were thawed and resuspended in humanized media comprised of Metabolomics AF media (custom formulation, InVitria) with 10 µg/mL human plasma low-density lipoproteins (EMD Millipore), 1% Penicillin/Streptomycin (VWR), and 2 ng/mL human IL6 (PeproTech). Samples were plated at 90,000 MNCs in 200 µL total volume per well in 96-well plates. They were then incubated ± drug at 37°C for 48 hours. Percent multiple myeloma cell viability was calculated by normalizing mean live treated multiple myeloma cell events to mean live untreated control events (15). SAR442257 and nullCD28 control were provided by Sanofi. Dara and Isa were obtained from the University of Colorado Health Pharmacy. Drug concentrations were chosen as the minimal dose that elicits the maximum reduction of viable primary multiple myeloma (15).

### Flow Cytometry

Samples were washed in FACS Buffer (PBS with 2% FBS), incubated with Brilliant Buffer (BD) and FcR block (Miltenyi Biotec) for 5 minutes, and stained with either a Myeloma panel: multi-epitope anti-CD38-FITC (Cytognos) chosen to avoid masking from clinically administered antibodies and SAR442257 treatment *ex vivo* (Supplementary Fig. S1), anti-CD138-BV421 MI15, anti-CD45-BV510 HI30, anti-BCMA-PE 19F2 (BioLegend), anti-CD46-APC E4.3, anti-CD56-PerCP-Cy5.5 B159, anti-CD19-BV605 SJ25C1, anti-CD28-APC-R700 CD28.2, and LIVE/DEAD Fixable Near-IR Stain (Thermo Fisher Scientific) or non-multiple myeloma (non-MM) panel: anti-CD3-FITC, anti-CD4-BV421, anti-CD107a-PE H4A3, anti-CD107b-PE H4B4, anti-CD28- APC CD28.2, anti-CD38-PerCP-Cy5.5, anti-CD16-BV510, anti-CD8-BV605, anti-CD56-APC-R700 NCAM16.2, and LIVE/DEAD Fixable Near-IR Stain (Thermo Fisher Scientific) for 10 minutes. Multiple myeloma cells are classified as live, singlet, CD38^+^CD138^+^ and non-MM cells are gated as live, singlet, CD38^−^CD138^−^ (Supplementary Fig. S1). All antibodies were from BD except when noted otherwise.

### CD3^+^ Cell Reduction

Primary multiple myeloma or human MNCs were thawed and washed in PBS. Human CD3 MicroBeads were used to remove CD3^+^ T cells from MNCs according to manufacturer's protocol (Miltenyi Biotec). Cells were run through MS Columns (Miltenyi Biotec) twice to ensure thorough depletion of CD3^+^ cells. MNCs were cultured with or without 1 nmol/L SAR442257 for 48 hours and Myeloma Drug Sensitivity Testing (My-DST) was performed.

### Statistical Analysis

Both statistics and figures were generated using GraphPad Prism. Data are presented as mean and SD and all patient sample experiments were performed in technical triplicate. When more than two means were compared, ANOVA with Tukey multiple comparisons was used to determine significance. Two-tailed Student *t* test was used when comparing two means. *P* values are labeled as *, *P* < 0.05; **, *P* < 0.01; ***, *P* < 0.001; and ****, *P* < 0.0001.

### Availability of Data and Material

Please send requests to daniel.sherbenou@cuanschutz.edu.

## Results

**FIGURE 1:**
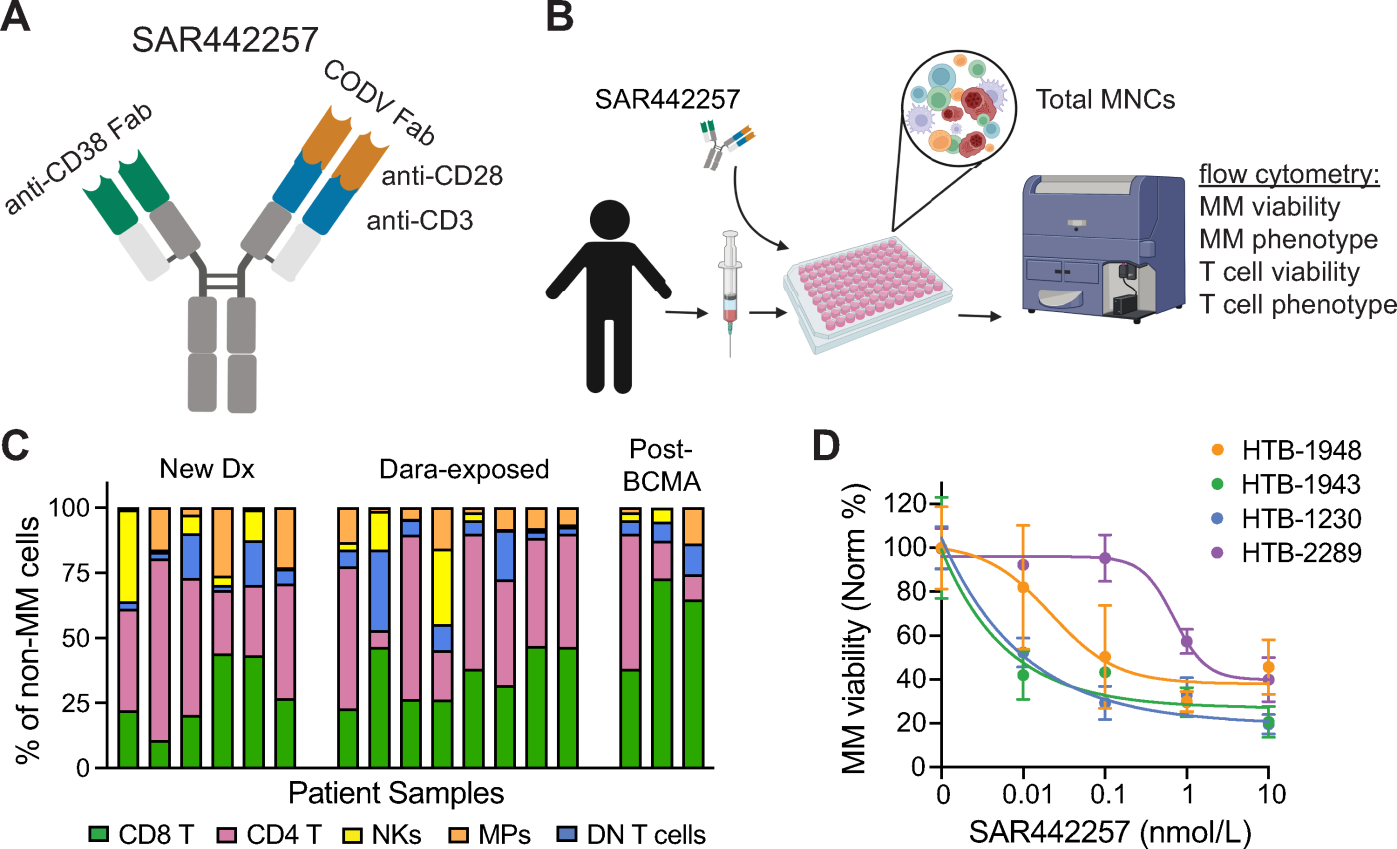
SAR442257 is a CD38/CD28xCD3 trispecific T-cell engager with measurable killing. **A,** Structure of SAR442257. **B,** My-DST for SAR442257 protocol. **C,** Immune composition (MM cells and B cells excluded) of My-DST cultures. **D,** Titrations of SAR442257 in three newly diagnosed multiple myeloma patient samples (HTB-1948, HTB-1943, HTB-1230) and 1 Dara-exposed (HTB-2289). Sensitive patient samples shown with nonlinear regression curve fitting. DN T cells, double-negative T cells; HTB, hematology tissue bank number; MM, multiple myeloma; MPs, macrophages; NKs, natural killer cells; Norm, normalized to controls.

SAR442257 is a trispecific antibody comprised of a CD28xCD3 cross-over dual variable fragment antibody region (Fab) opposing an α-CD38 Fab (Fig. 1A; ref. 10). We sought to examine the activity of this anti-CD38 TCE in patient BM aspirates of various treatment histories using their own immune effector cells. To achieve this, we utilized My-DST, consisting of 48 hours *ex vivo* culture of total peripheral blood or BM MNCs in humanized media followed by multiparameter flow cytometry to assess multiple myeloma cell specific survival and phenotype (Fig. 1B and C; ref. 15). As total MNCs from primary aspirates include both multiple myeloma and T cells, we are able to directly measure TCE activity using the patients’ own T cells, which in multiple myeloma are often from heavily treated patients of advanced age. To identify the dose for screening at a single concentration to judge sensitivity versus resistance, we performed dose–response titrations on samples from 6 patients. SAR442257 was potent with EC_50_s in the picomolar range (Fig. 1D). As My-DST primarily seeks to identify sensitivity, the approximate EC90 of 1 nmol/L as the minimal dose of maximum effect to perform further mechanistic studies and screen additional samples from different disease settings (14, 15).

**FIGURE 2:**
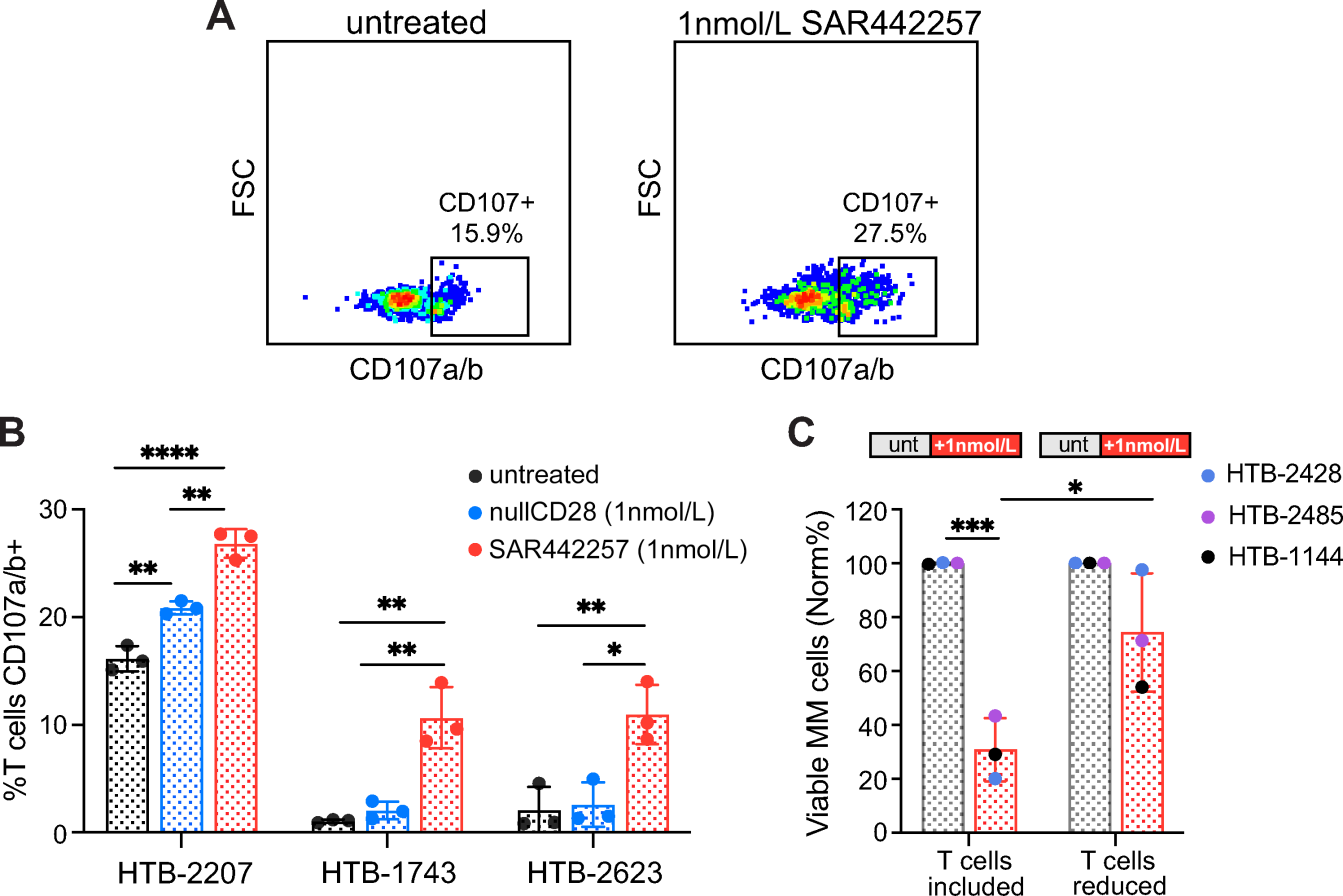
SAR442257 induces T cell–mediated multiple myeloma (MM) cell cytotoxicity that is superior to dual targeting antibodies. **A,** Representative example of a sample stained and gated for CD107a/b to show degranulation of T cells in MNC cultures treated with 1 nmol/L SAR442257 versus untreated control. **B,** Results from three multiple myeloma patient samples showing higher degranulation of CD3^+^ T cells in the presence of 1 nmol/L SAR442257 compared with 1 nmol/L nullCD28 controls or untreated controls. Statistical significance by one-way ANOVA with Tukey multiple comparisons. **C,** Efficacy of SAR442257 in My-DST was significantly less with CD3^+^ T cells reduced by magnetic bead separation from three multiple myeloma patient samples prior to 48 hours treatment with 1 nmol/L SAR442257. Each color represents a biological replicate calculated as the average of three technical replicates. Statistical significance by unpaired *t* test. *, *P* < 0.05; **, *P* < 0.01; ****, *P* < 0.0001. HTB, hematology tissue bank number.

Next, we sought mechanistic insight into the anti-MM cell cytotoxicity of SAR442257 in My-DST. SAR442257 induced degranulation of primary endogenous T cells in culture, as measured by surface CD107a and CD107b on CD3^+^ T cells with or without drug (Fig. 2A). We observed significantly higher surface CD107a and CD107b expression in BM MNCs from patients treated with 1 nmol/L SAR442257, demonstrating that multiple myeloma cell killing occurs in concert with T-cell degranulation (Fig. 2B). Trispecific CD38/CD28xCD3 engagement also appears to induce more degranulation than bispecific CD38xCD3 engagement. To demonstrate this, we used a null-mutant CD28 control, which contains a knockout mutation in the anti-CD28 F_v_ to prevent binding, but otherwise identical to SAR442257. BM MNCs were cultured with media, SAR442257, or null-mutant CD28. CD107a and CD107b surface expression on T cells was significantly higher in BM MNCs treated with SAR442257 than with null-mutant CD28 in all three patient samples (Fig. 2B). To further confirm activity through endogenous patient cells, we removed T cells from patient BM MNCs and performed My-DST with or without SAR442257. BM MNCs were significantly less sensitive to SAR442257 when T-cell number was reduced in BM MNCs before culture, supporting that the anti-multiple myeloma cell cytotoxicity of SAR442257 in My-DST cultures is T cell mediated (Fig. 2C). These data confirm that SAR442257 cytotoxicity is T cell dependent and show that dual CD28 and CD3 engagement accentuates T-cell activity.

**FIGURE 3:**
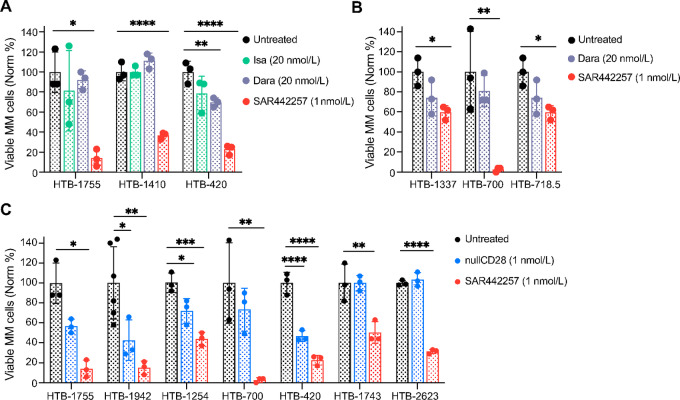
SAR442257 is more effective against primary multiple myeloma (MM) than single or dual targeting antibodies. **A,** The efficacy of the minimum effective concentration of SAR442257 was significantly more effective than that of Dara or Isa in three Dara-exposed (HTB-1775, HTB-1410, and HTB-420) patient aspirates after 48 hours culture measured by My-DST. **B,** The efficacy of the minimum effective concentration of SAR442257 was significantly more effective than Dara in three post-CAR-T (HTB-1337, HTB-718, and HTB-700) patient aspirates after 48 hours culture measured by My-DST. **C,** SAR442257 was significantly more efficacious than null-mutant CD28 control in one NDMM (HTB-2623), five Dara-exposed (HTB-1755, HTB-1942, HTB-1254, HTB-420, and HTB-1743), and one Dara-exposed, post-BCMA (HTB-700) patient aspirate. Technical triplicates of each biological replicate are shown in the same color. Statistical significance for A–C were calculated by one-way ANOVA with Tukey multiple comparisons. *, *P* < 0.05; **, *P* < 0.01; ***, *P* < 0.001; ****, *P* < 0.0001. Dara, daratumumab; Isa, isatuximab.

We next tested whether T cell–engaging SAR442257 yields greater multiple myeloma cell cytotoxicity than the CD38 antibodies that are important components of current clinical care. We previously established 20 nmol/L as the minimal concentration of maximum effect for screening experiments for both mAbs (14, 15). We performed My-DST on 6 patients previously exposed to Dara, 3 of which were also post-BCMA CAR-T. The EC90 of 20 nmol/L was used to measure Isa and Dara sensitivity. These EC90s have been previously identified as the minimal dose of maximum effect using dose titrations in anti-CD38 mAb naïve patients (14, 15). Primary multiple myeloma cells were more sensitive to treatment with 1 nmol/L SAR442257 than Dara or Isa, despite their experimental EC90s being 20-fold that of SAR442257 (Fig. 3A and B). As mentioned above, SAR442257 is modified from the prototypical TCE format by the inclusion of a CD28 binding domain. Thus, we performed My-DST in six primary samples comparing 1 nmol/L SAR442257 and 1 nmol/L null-mutant CD28 control. SAR442257 showed greater multiple myeloma cell cytotoxicity than the typical bispecific null-mutant CD28 antibody (Fig. 3C). These findings demonstrate the advantages of trispecific antibody engagement, yielding better anti-MM cell cytotoxicity compared to bispecific TCEs or mAbs.

**FIGURE 4:**
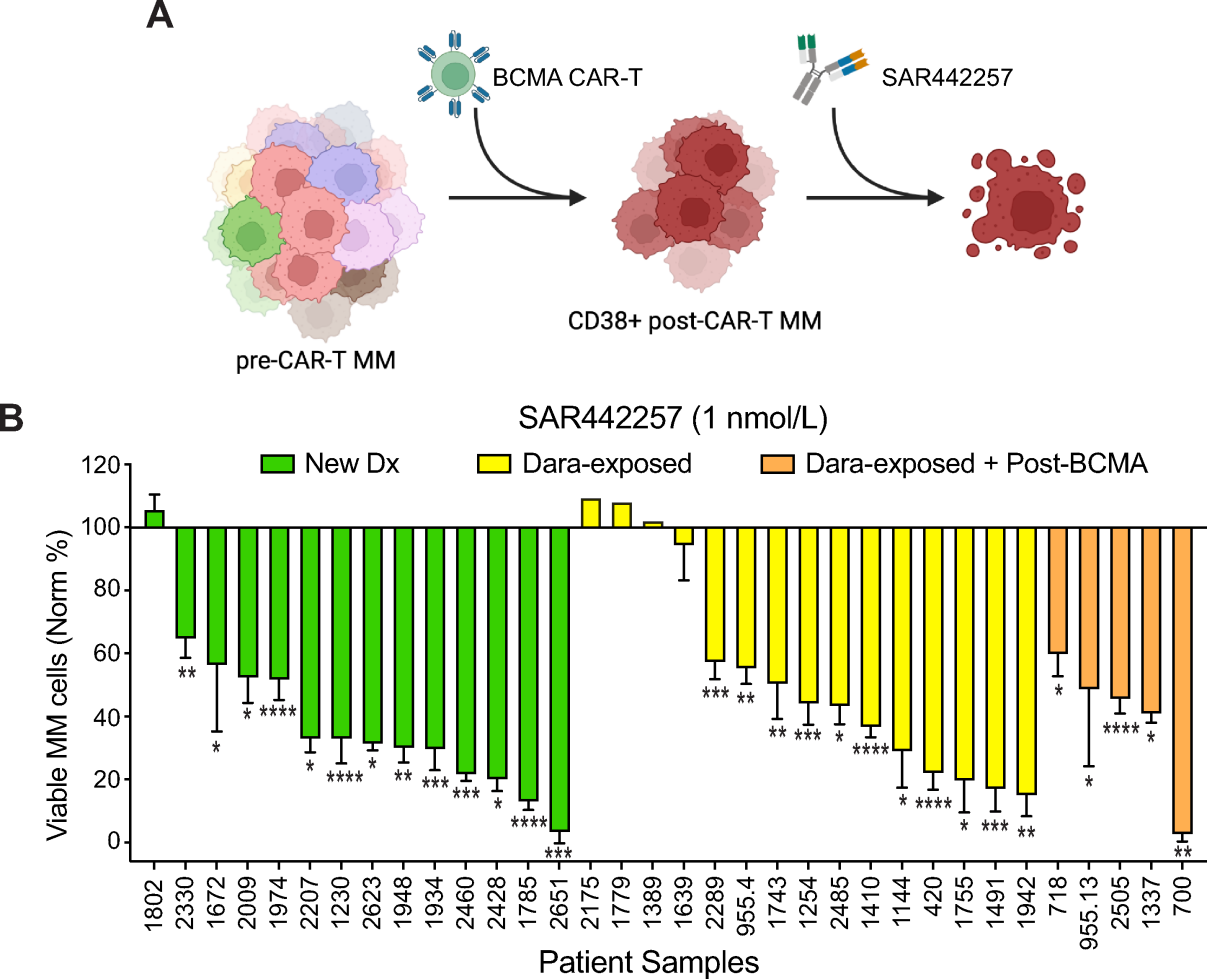
SAR442257 is effective across disease settings including relapse after CAR-T. **A,** We hypothesized that CD38 recovers in Dara-exposed, post-CAR-T multiple myeloma (MM) cells and is sensitive to targeting with SAR442257. **B,** Efficacy of SAR442257 (1 nmol/L) in 14 NDMM, 15 Dara-exposed, and five Dara-exposed, post-BCMA primary aspirates after 48 hours culture measured by My-DST. HTB-2505 is post-belanatamab mafodotin; the remaining post-BCMA samples are post-BCMA CAR-T. Mean and SD of three technical replicates. Sensitivity to SAR442257 is indicated statistical significance, analyzed by one-way ANOVA or by unpaired *t* test according to experimental setup. *, *P* < 0.05; **, *P* < 0.01; ***, *P* < 0.001; ****, *P* < 0.0001. CAR-T, chimeric antigen receptor T cells; Dara, daratumumab; Norm, normalized to untreated controls.

In our study, our main objective was to inform the clinical setting best suited for application of SAR442257. As our previous work has shown that patients have better *ex vivo* response to anti-CD38 retreatment after increasing amounts of time off of CD38-targeting agents, we hypothesized that SAR442257 would be effective in patients relapsed after anti-CD38 mAbs and BCMA therapy (Fig. 4A; ref. 14). In total, we screened 34 primary aspirates for sensitivity to SAR442257. Effect of SAR442257 on multiple myeloma cell viability was determined by normalizing the number of remaining multiple myeloma cells following SAR442257 My-DST to untreated control wells. These aspirates were taken from newly diagnosed multiple myeloma (NDMM), Dara-exposed, and Dara-exposed + post-BCMA therapy patients. Patients were of diverse treatment history and cytogenetic status (Supplementary Table S1). Dara-exposed patients underwent a washout period from Dara that averaged 37 weeks, with a median of 12 weeks (Supplementary Table S1). Of these, 85% (29/34) of primary aspirates were sensitive to 1 nmol/L SAR442257 (Fig. 4B; Supplementary Fig. S2A). More patients with NDMM were sensitive (93%, 13/14 patients) than Dara-exposed patients (80%, 16/20 patients), although there was no statistically significant difference in depth of response between these groups (Supplementary Fig. S2B). Of the Dara-exposed aspirates, five of five (100%) samples that were also post-BCMA were sensitive to SAR442257. This supports clinical testing of the anti-CD38 SAR442257, with good potential for response in patients who relapse after BCMA therapy.

**FIGURE 5:**
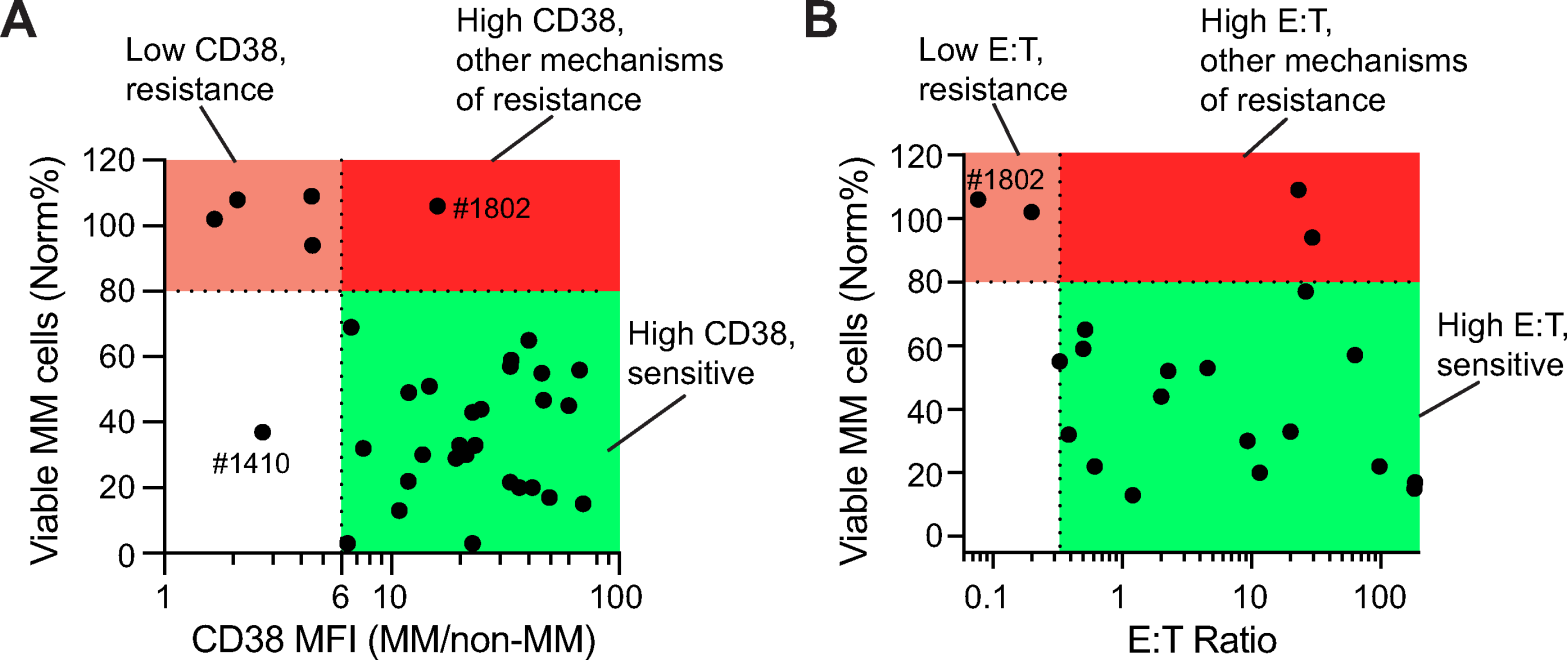
Biomarkers of *ex vivo* response to anti-CD38 T-cell engager. **A,** The ratio of geometric MFI of CD38 on multiple myeloma (MM) cells normalized to CD38 on CD38^−^CD138^−^ non-MM cells is 6-fold higher in patient aspirates with a significant response to SAR442257 (1 nmol/L) after 48 hours culture. Sensitivity cutoff is indicated by horizontal dashed line, as all primary aspirates with < 80% viable multiple myeloma cells after treatment showed statistically significant sensitivity and has been previously shown to correlate with response (15). Post-CAR-T biopsies are labeled in italics. HTB-1802 is the only primary aspirate that failed to respond despite high CD38 expression relative to non-MM cells. HTB-1410 is the only aspirate sensitive to SAR442257 despite low CD38 expression relative to non-MM cells. **B,** Effector-to-target ratio in My-DST culture, calculated as number of CD3^+^ cells divided by number of CD138^+^CD38^+^ cells per untreated well. Sensitivity cutoff is indicated by horizontal dashed line, as all aspirates with <80% viable multiple myeloma cells after treatment showed statistically significant sensitivity and has been previously shown to correlate with response (15). E:T, effector-to-target ratio; Norm, normalized to untreated controls.

As SAR442257 showed occasional cases of resistance in both NDMM and Dara-exposed patients, we next sought to investigate potential biomarkers of response to SAR442257. First, we examined CD38 target expression by dividing the mean fluorescence intensity (MFI) of CD38 on CD38^+^CD138^+^ multiple myeloma cells by that of CD38^−^CD138^−^ non-MM cells. We found that primary multiple myeloma cells sensitive to SAR442257 had a CD38 MFI 6-fold brighter than the surrounding non-MM cells in culture, with the exception of two samples, HTB-1410 and HTB-1802 (Fig. 5A). Of note, all five post-BCMA patient multiple myeloma samples (HTB-700, HTB-718, HTB-955.13, HTB-2505, and HTB-1337) had CD38 expression more than 6-fold greater than on non-MM cells, supporting our hypothesis that post-Dara patients relapsed after BCMA therapy have sufficient CD38 expression to respond to SAR442257 (Supplementary Fig. S3). These findings suggest that as long as CD38 MFI is 6-fold brighter on multiple myeloma cells relative to surrounding non-MM cells, SAR442257 has potent anti-MM cell activity. This is similar to previous findings reported with Dara, and further analysis in a clinical trial setting could be used to establish CD38 expression as a biomarker of SAR442257.

Next, we sought to identify factors other than CD38 expression that may associate with response to SAR442257. First, we examined the relationship between T-cell number and phenotype and response to SAR442257. In our study, we found no correlation between effector-to-target (E:T) ratio (calculated as the no. of CD3^+^ T cells/no. of multiple myeloma cells per well, averaged between three technical replicates to compare between patient samples) and sensitivity to SAR442257, but these data do suggest that a low E:T ratio (< 0.33) may result in lack of response (Fig. 5B). This is illustrated by HTB-1802, the only sample resistant to SAR442257 with high multiple myeloma cell CD38 expression that contained a low E:T ratio. In our cohort, we saw no correlation between the abundance of CD4^+^ versus CD8^+^ T cells and sensitivity (Supplementary Fig. S4). As CD28 expression has been found on multiple myeloma cells, we examined whether the percent of multiple myeloma cells positive for CD28 affected response to SAR4422257. We saw no correlation between the percentage of multiple myeloma CD28^+^ cells and the sensitivity to SAR442257 in our cohort (Supplementary Fig. S5). Taken together, these data support that CD38 expression level is the strongest predictor of response to SAR442257, a phenomenon that occurs even in patients previously treated with anti-CD38 mAbs. Further measurement of E:T ratios in patients treated with bispecific antibodies may be another interesting clinical correlative biomarker that can influence response.

## Discussion

To our knowledge, this study is the first to examine the activity of a TCE using matched patient endogenous T cells in primary multiple myeloma cells. To leverage this approach, we sought to identify the most effective clinical setting for novel TCE SAR442257. On the basis of our previous findings, we hypothesized that RRMM recovers CD38 expression after time off Dara, allowing patients previously declared resistant to CD38-targeting to regain sensitivity (14). As expected, there was some resistance seen in patients who had received prior anti-CD38 mAbs, but still most Dara-exposed aspirates were sensitive. Therefore, while prior analysis shows that CD38 antibody retreatment becomes more successful the longer the washout period from last dose of Dara, a CD38 TCE appears less likely to have cross-resistance (13, 14). Furthermore, SAR442257 showed promise in a small number of aspirates available from patients relapsed after BCMA therapy, with all demonstrating sensitivity. Of these patients, 2/5 were receiving Dara as salvage therapy immediately post-BCMA CAR-T and at the time these aspirates were taken. Despite this, CD38 expression on multiple myeloma cells remained bright. This is consistent with our finding that of the 29/34 patient aspirates responding to SAR442257, and the most promising biomarker of response was high CD38 expression on multiple myeloma cells relative to surrounding non-MM cells.

CD38 is a well-established target in multiple myeloma, and effective CD38 retreatment strategies would be a timely development in the changing landscape of multiple myeloma treatment. BCMA CAR-T has shown a favorable survival benefit compared to other therapies available for heavily pretreated patients, and ongoing studies are examining whether CAR-T works even better in the newly diagnosed setting (2, 3, 16–18). Meanwhile, CD38 mAbs are more often being used in frontline treatment regimens (11, 19). As these effective therapies are given earlier in disease progression, researchers must address a future where most patients with RRMM are already CD38 and BCMA-exposed. While the optimal washout period from CD38 mAbs is debated, the observed CD38 recovery after exposure to CD38 mAbs and our data suggest retargeting CD38 with a TCE may be effective in Dara-exposed post-BCMA RRMM (14, 20, 21). In addition, a recent *post hoc* analysis has shown response to GPRC5DxCD3 T-cell engager talquetamab in 67% of those who had received prior BCMA CAR-T ide-cel and 83% of those who had received cilta-cel, suggesting that endogenous T-cell cytotoxicity is not significantly impacted by prior CAR-T therapy (7). However, further study in patients with RRMM is needed to confirm such response. Recent work has suggested that the pre-existing health of CD8^+^ T cells in patients with multiple myeloma determines TCE response, with a high abundance of exhausted-like CD8^+^ T cells predicting TCE resistance (22). As T-cell exhaustion increases with myeloma progression, future analyses should examine the relationship between T-cell phenotype and proliferation, disease state, and sensitivity to TCEs in multiple myeloma (23–24). SAR442257 is currently being evaluated in a phase I clinical trial (ClinicalTrials.gov #NCT04401020), results pending.

TCEs against antigens other than BCMA and CD38 are also showing promise, exemplified by the recent approval of talquetamab (7, 25). Here, we used My-DST to demonstrate the potent killing of primary multiple myeloma cells by SAR442257 using the patients’ own endogenous effector cells. While the E:T ratio of endogenous T cells to multiple myeloma cells did not correlate with sensitivity in our study, further clinical study of E:T ratio and TCE response would be interesting to see whether this does influence response. With the recent approval of three TCEs for RRMM, clinicians are given little guidance on sequencing of T-cell therapies. *Ex vivo* sensitivity measured by My-DST has been previously shown to correlate with clinical response (15). Use of a personalized approach examining *ex vivo* drug sensitivity and/or target expression levels with matched primary T cells like My-DST could prove a valuable tool to inform the order of TCE regimens for each patient going forward. A phase II clinical trial is underway examining the correlation between My-DST results and clinical outcome to nuclear export inhibitor Selinexor (ClinicalTrials.gov #NCTNCT04925193), results pending.

## Supplementary Material

Supplementary Figure 1 Multi-epitope CD38-FITC binds CD38 in cells treated with SAR442257.

Supplementary Figure 2 Sensitivity to SAR4422557 across treatment groups

Supplementary Figure 3 CD38 expression across treatment groups

Supplementary Figure 4 Ratio of CD8+ to CD4+ T cells does not appear to affect sensitivity to SAR442257

Supplementary Figure 5 The percentage of MM cells CD28+ does not appear to affect sensitivity to SAR442257

Supplementary Table 1 Treatment History of Patients Donating Samples
