# Supplementary material for: Ex Vivo Efficacy of SAR442257 Anti-CD38 Trispecific T-cell Engager in Multiple Myeloma Relapsed After Daratumumab and BCMA-targeted Therapies
Source: Cancer Res Commun. 2024 Mar 12;4(3):757–64. doi: 10.1158/2767-9764.CRC-23-0434 (PMC10929583; doi:10.1158/2767-9764.CRC-23-0434)
Supplement: Supplementary Figure 1 — Multi-epitope CD38-FITC binds CD38 in cells treated with SAR442257. [file crc-23-0434-s01.docx]

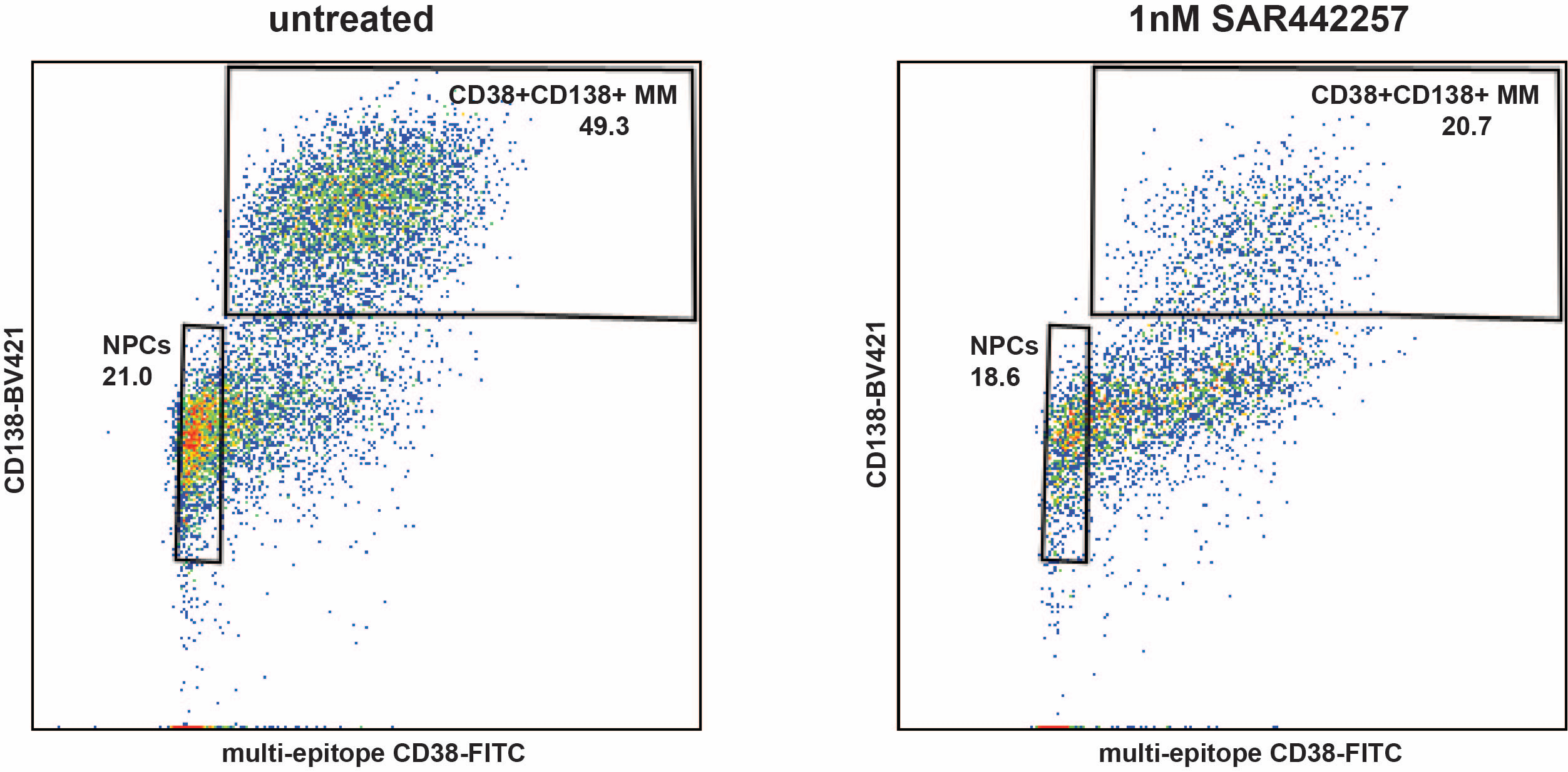


**Non-MM**

**Non-MM**

**Supplemental Figure 1. Multi-epitope CD38-FITC binds CD38 in cells treated with SAR442257.** CD38+CD13+ MM gating of live cells in HTB-1230 (NDMM). Multi-epitope CD38-FITC binds CD38 in cells treated with SAR442257.
