# Supplementary material for: Ex Vivo Efficacy of SAR442257 Anti-CD38 Trispecific T-cell Engager in Multiple Myeloma Relapsed After Daratumumab and BCMA-targeted Therapies
Source: Cancer Res Commun. 2024 Mar 12;4(3):757–64. doi: 10.1158/2767-9764.CRC-23-0434 (PMC10929583; doi:10.1158/2767-9764.CRC-23-0434)
Supplement: Supplementary Figure 2 — Sensitivity to SAR4422557 across treatment groups [file crc-23-0434-s02.docx]

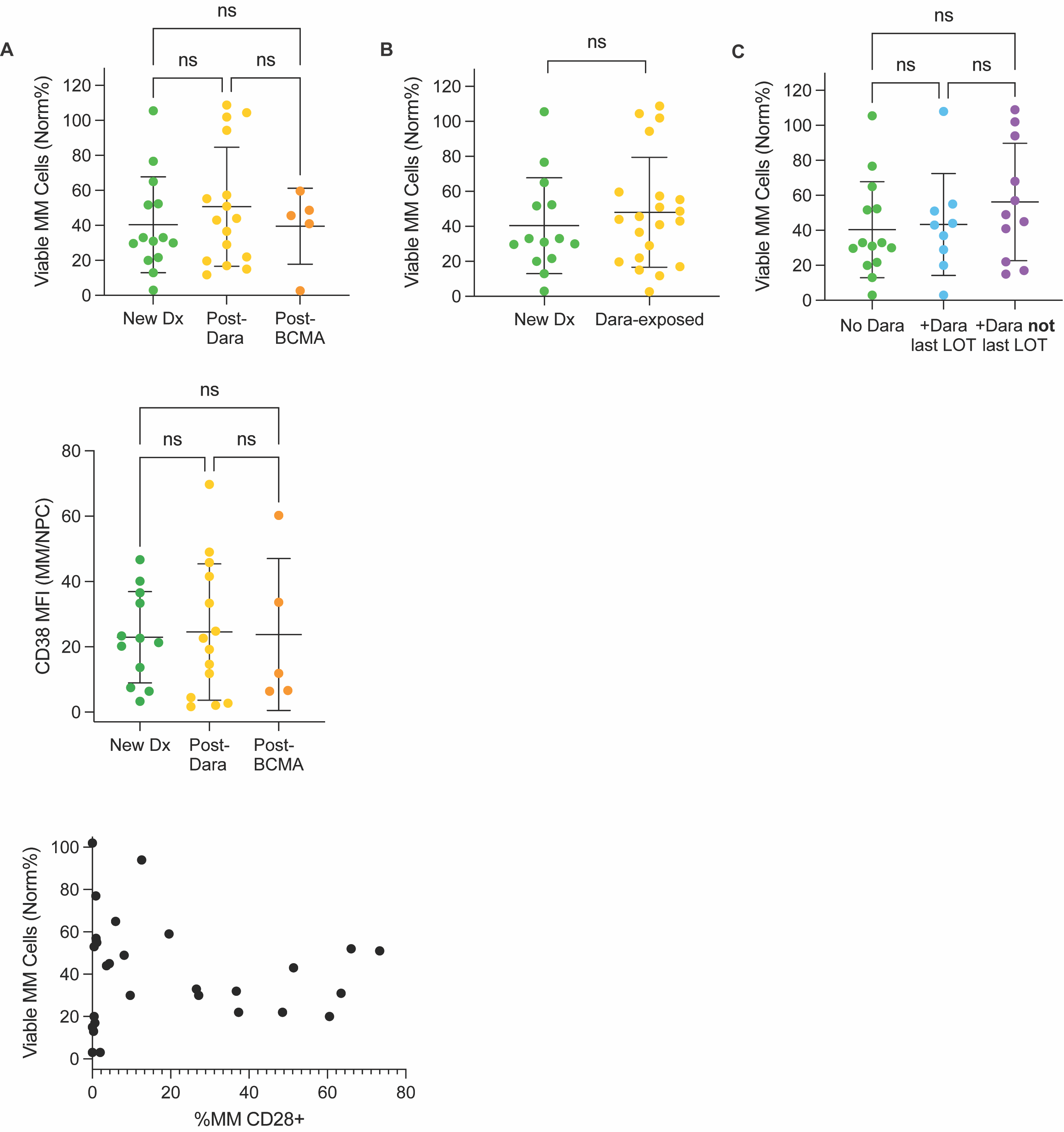

**Supplemental Figure 2. Sensitivity to SAR4422557 across treatment groups.** (A-B) Sensitivity to SAR442257 organized by treatment setting following 48 hr culture with 1nM SAR442257 organized by NDMM, Post-Dara, and Post-BCMA (A) or NDMM vs. Dara-exposed (Post-Dara+Post-BCMA) (B). (C) Sensitivity to 1 nM SAR442257 in daratumumab (Dara) naiive patients, patients who received Dara as their last line of therapy (LOT) before their biopsy was taken, and patients who received Dara but not as their most recent LOT. (A-B) Analyzed by Kruskal-Wallis test with multiple comparisons.
