# Supplementary material for: Ex Vivo Efficacy of SAR442257 Anti-CD38 Trispecific T-cell Engager in Multiple Myeloma Relapsed After Daratumumab and BCMA-targeted Therapies
Source: Cancer Res Commun. 2024 Mar 12;4(3):757–64. doi: 10.1158/2767-9764.CRC-23-0434 (PMC10929583; doi:10.1158/2767-9764.CRC-23-0434)
Supplement: Supplementary Figure 3 — CD38 expression across treatment groups [file crc-23-0434-s03.docx]

**
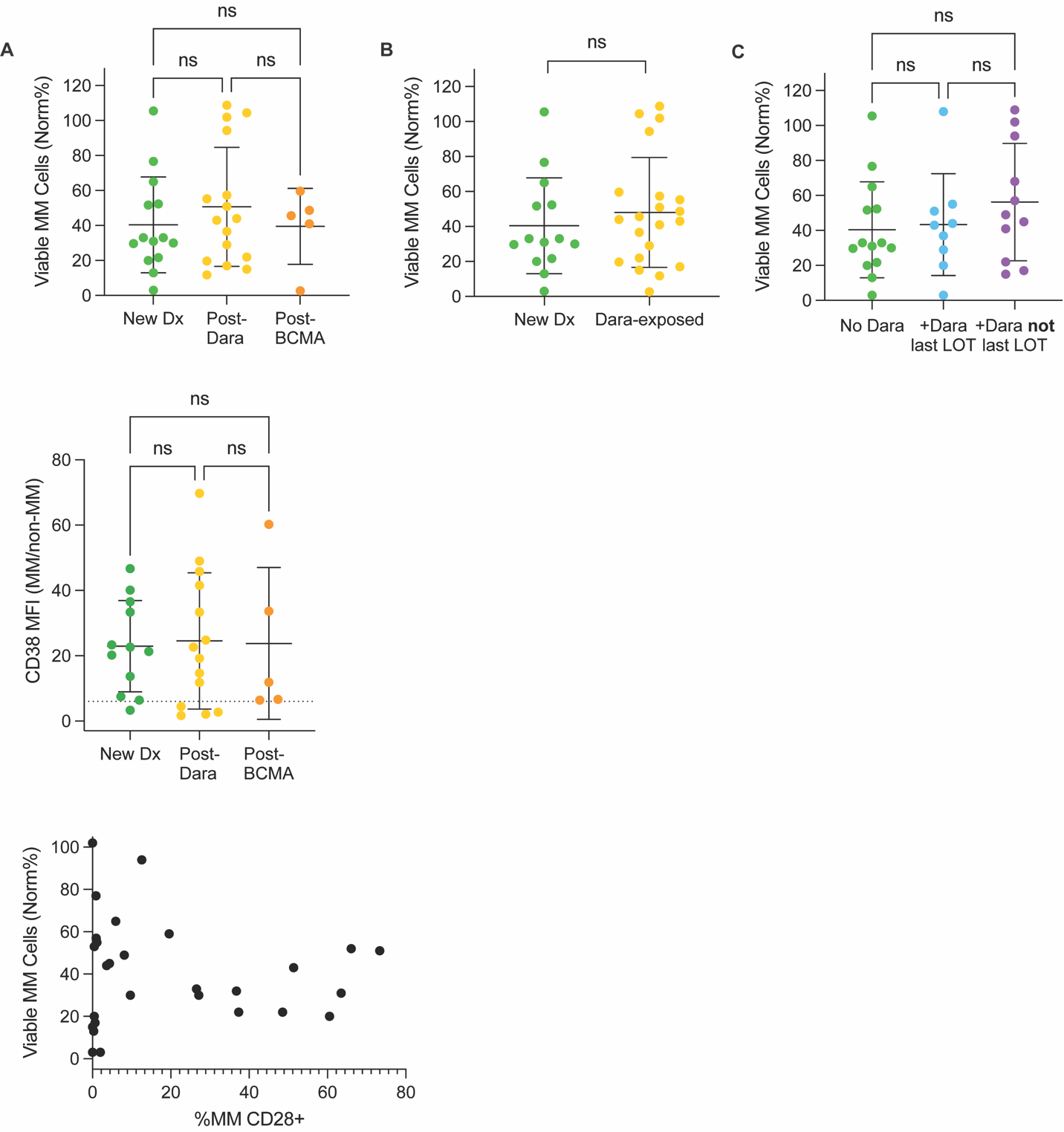
**

**Supplemental Figure 3. CD38 expression across treatment groups.** CD38 MFI on CD38+CD138+ MM cells divided by CD38 MFI on CD38-CD138- non-MM cells for newly diagnosed, Daratumumab-exposed, and post-CAR-T patient biopsies. Dash line indicates a CD38 MFI MM/non-MM ratio of 6. Each dot represents the average of three technical replicates.
