# Supplementary material for: Ex Vivo Efficacy of SAR442257 Anti-CD38 Trispecific T-cell Engager in Multiple Myeloma Relapsed After Daratumumab and BCMA-targeted Therapies
Source: Cancer Res Commun. 2024 Mar 12;4(3):757–64. doi: 10.1158/2767-9764.CRC-23-0434 (PMC10929583; doi:10.1158/2767-9764.CRC-23-0434)
Supplement: Supplementary Figure 5 — The percentage of MM cells CD28+ does not appear to affect sensitivity to SAR442257 [file crc-23-0434-s05.docx]

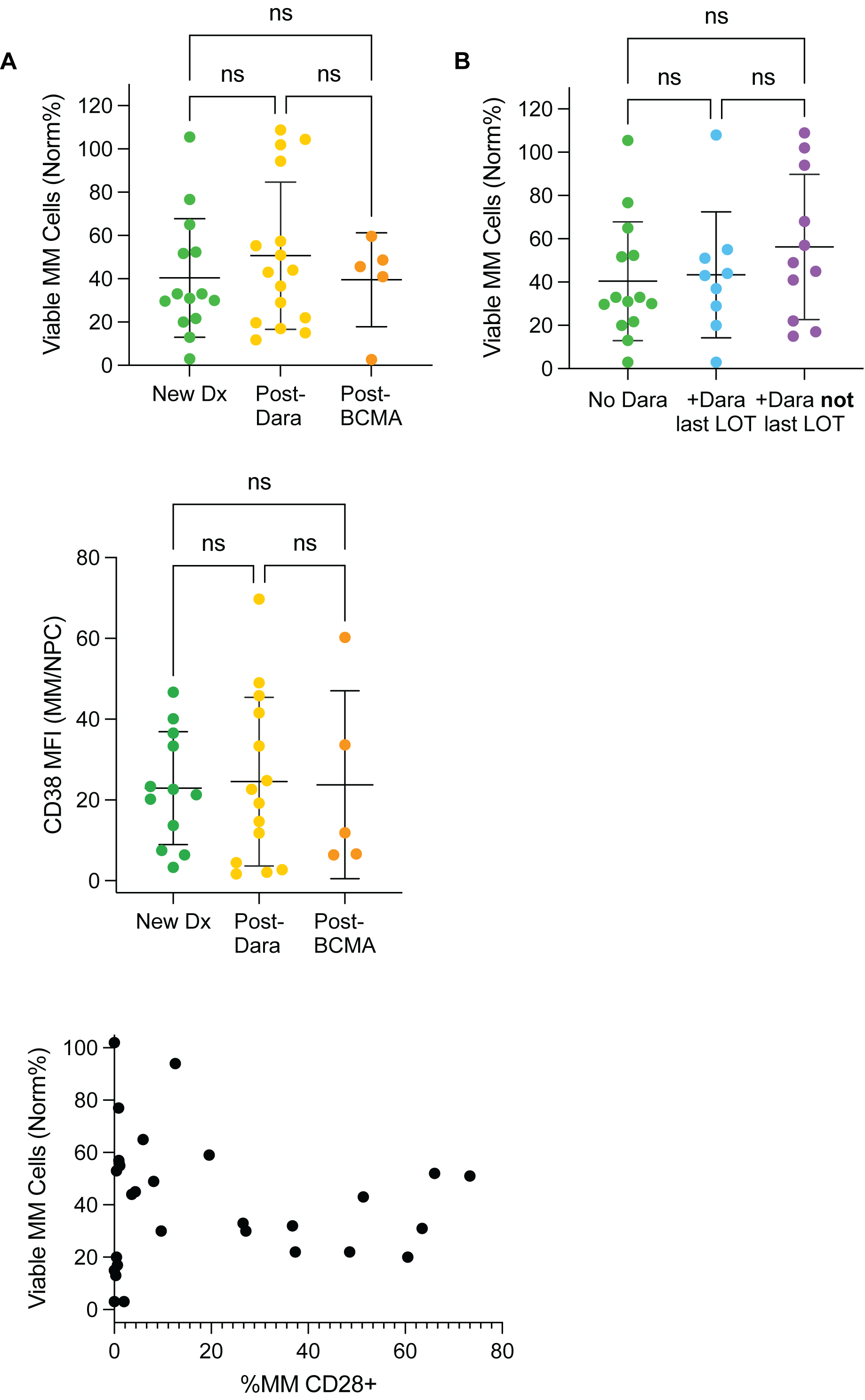


**Supplemental Figure 5. The percentage of MM cells CD28+ does not appear to affect sensitivity to SAR442257.** Percent of total untreated MM cells that are positive for surface CD28 expression vs. MM cell viability following treatment with 1 nM SAR442257. Each dot represents the average of three technical replicants for an individual patient aspirate. Analyzed by simple linear regression.
