# Supplementary material for: Ex Vivo Efficacy of SAR442257 Anti-CD38 Trispecific T-cell Engager in Multiple Myeloma Relapsed After Daratumumab and BCMA-targeted Therapies
Source: Cancer Res Commun. 2024 Mar 12;4(3):757–64. doi: 10.1158/2767-9764.CRC-23-0434 (PMC10929583; doi:10.1158/2767-9764.CRC-23-0434)
Supplement: Supplementary Table 1 — Treatment History of Patients Donating Samples [file crc-23-0434-s06.docx]

**Supplemental Table 1. Treatment History of Patients Donating Samples.**

| **Patient Number** | **Sample Type** | **FISH** | **Prior Treatment Lines in Order Received** | **Time off Dara** | **%MM cell Survival after 1nM SAR442257** |
| --- | --- | --- | --- | --- | --- |
| HTB-1802 | BM | unknown | None | N/A | 106 |
| HTB-2330 | BM | gain 5p15, 9q22, 15q22 | None | N/A | 65 |
| HTB-1974 | BM | TP53 | None | N/A | 52 |
| HTB-2207 | BM | unknown | None | N/A | 33 |
| HTB-1230 | BM | Hyperdip, 1q+, 13q- | None | N/A | 33 |
| HTB-2623 | BM | unknown | None | N/A | 31 |
| HTB 1672 | BM | 17+, 11q+, 20q+ | None | N/A | 56 |
| HTB-1948 | BM | Iq21 gain, 15q22 gain, del 13q14, del 13q34, monosomy 13 | None | N/A | 30 |
| HTB-1934 | BM | 5p15/9q22/15q22, trisomy 5, 9, 15; del 13q14, 13q34, monosomy 13, | None | N/A | 30 |
| HTB-2460 | PB | gain 1q21, 1p32, 5p15, 9q22, 15q22, tetrasomy tp53, IGH:MAF rearrangement, gain IGH | None | N/A | 22 |
| HTB-2428 | BM | gain 1q21, 5p15, 9q22, 15q22, del 13q14, 13q34, IGH rearrangement, TP53, 17q11 | None | N/A | 20 |
| HTB-1785 | BM | 17p-, 11q+ | None | N/A | 13 |
| HTB-2651 | BM | Unknown | None | N/A | 3 |
| HTB-2009 | BM | gain of 4p16, 5p15, 9q22, 11q13, and 15q22 sequences | None | N/A | 53 |
| HTB-2175 | BM | gain 11q13 | VRD, ASCT, mR, DaraKD, Dmul | 32 weeks | 109 |
| HTB-1779 | PB | t(11;14) del17p | CyBorD, ASCT, mRID, DarKD | 7 weeks | 108 |
| HTB-1389 | PB | t(4;14), 17p-, 1p-, 6q-, 13- | VD, R, Mul, DKDara, CyVD | 7 weeks | 102 |
| HTB-1639 | BM | Hyperdip, 1q+, trisomy 13, 17p+, 4p+, 11q+ | VDR, DaraPD, CyKD, anti-CD74 antibody trial, VD-Mul, ASCT | 60 weeks  (Approx 1.1 years) | 94 |
| HTB-2289 | BM | loss of TP 53, low level gains of 9q22.15q22 ,and IGH | RVD, DaraRD, DaraID, DPElo, CyKD | 17 weeks | 57 |
| HTB-0955.4 | BM | gain 1q21, 5p15, 9q22, 15q22, del 13q14, 13q34, | ASCT, DTh, RD, KRD, PD, DaraKD | 5 weeks | 55 |
| HTB-1743 | BM | Gain 12q, 16p, 22q, 18q | CyVD, DaraVRD, ASCT, DaraRD | 5 weeks | 51 |
| HTB-1254 | BM | Hyperdip (1q+, trisomy 11, monosomy 13, 17p+) | RVD, KRD, DarPD, DARPID | 24 weeks | 44 |
| HTB-2485 | BM | Unknown | CyVD, DaraVR | 4 weeks | 43 |
| HTB-1410 | PB | normal | VRD, ASCT, DarRD | 5 weeks | 37 |
| HTB-1144 | BM | Previous gain 1p32, 1q21, tp15, 9q22,15q22 | CyVD, ASCT, V, KRD, DaraD, CyKD, IsaKD, Cevostamab, Dara-CyVRD | 3 weeks | 29 |
| HTB-0420 | BM | Previous CDKN2C, CKS1B, gain 1q, 15q22, del 13q14 and 13q34 | CyVD, KRD, ASCT, K, VRD, DaraDPom, Dar/VD, Cevostamab | 64 weeks  (Approx 1.2 years) | 22 |
| HTB-1755 | BM | t(8;14), 11q+ | CyVD, mRD, KRD, ASCT, mRev, EloRD, DaraVD | Currently on Dara | 20 |
| HTB-1491 | BM | Previous  1q+, 13q-, 20q- | RVD, R, DaraVD, ASCT | 184 weeks  (Approx 3.5 years) | 17 |
| HTB-1942 | BM | Previous t(11;14), gain1q | VThD-Mul, ASCT, DarVD, RBD | 16 weeks | 15 |
| HTB-0718 | BM | del 20q, low level IGH/CCND1 and 1q21 | CyVD, KRevD, ASCT, DarKD,Car-T, CD46 antibody drug conjugate Trial | 33 weeks | 68 |
| HTB-0955.13 | BM | Previous gain 11q13 | Induction unknown, ASCT, ThD, RevD, KRD, PD, DaraKDex, CAR-T | 88 weeks  (Approx 1.7 years) | 49 |
| HTB-2505 | BM | Unknown | RVD, CyVD, ASCT, R, Elo/Re, DaraVD, KPD, Vel/D, Belantamab mafodotin | 124 weeks  (Approx 2.5 years) | 45 |
| HTB-1337 | BM | gain 1q21, gain 5p15, 9q22, 15q22, del 13q14 and 13q34, del tp554, IGH/FGFR3 rearrangment, tetrasomy for 11q13 (CCND1) and IGH, IGH rearrangement | CyVD, Mel-ASCT,mR, EloRD, EloPD, KPD, DaraVRD, CAR-T x2, DarSelD | 4 weeks | 41 |
| HTB-0700 | PB | 13q14, IGH-CCND1 | CyVD, SCT, RD, VD-venetoclax trial, DaraPD, CAR-T, DaraKD | 8 weeks | 3 |

ASCT, autologous stem cell transplant with melphalan; BM, bone marrow biopsy; CAR-T, BCMA chimeric antigen receptor T cell therapy; Cy, cyclophosphamide; D, dexamethasone; Dara, daratumumab; Elo, elotuzumab; I, ixazomib; Isa, isatuximab, K, carfilzomib; m, maintenance; Mul, multiple agent chemotherapy; Ob, observation; P, pomalidomide; PB, peripheral blood; R, Revlimid (lenalidomide); Sel, Selinexor, Th, Thalidomide; Unk, unknown; V, Velcade (bortezomib)
